# High Prevalence of Sexually Transmitted and Reproductive Tract Infections (STI/RTIs) among Patients Attending STI/Outpatient Department Clinics in Tanzania

**DOI:** 10.3390/tropicalmed8010062

**Published:** 2023-01-13

**Authors:** Said Aboud, Simon N. Buhalata, Onduru G. Onduru, Mercy G. Chiduo, Gideon P. Kwesigabo, Stephen E. Mshana, Alphaxard M. Manjurano, Mansuet M. Temu, Coleman Kishamawe, John M. Changalucha

**Affiliations:** 1 Departments of Microbiology and Immunology, Epidemiology and Biostatistics, Muhimbili University of Health and Allied Sciences (MUHAS), Dar es Salaam P.O. Box 65001, Tanzania; 2 National Institute for Medical Research, Mwanza Research Centre, Mwanza P.O. Box 1462, Tanzania; 3 The Africa Center of Excellence in Public Health and Herbal Medicine (ACEPHEM), Kamuzu University of Health Sciences, Blantyre P.O. Box 360, Malawi; 4 National Institute for Medical Research, Tanga Research Centre, Tanga P.O. Box 5004, Tanzania; 5 Department of Medical Microbiology, Catholic University of Health and Allied Sciences (CUHAS), Mwanza P.O. Box 1370, Tanzania

**Keywords:** *N. gonorrhoeae*, *C. trachomatis*, STI/RTIs, bacterial vaginosis, vaginal candidiasis

## Abstract

We determined the prevalence and reported risk factors associated with sexually transmitted and reproductive tract infections (STI/RTIs) among patients who presented with genital symptoms in STI/outpatient department (OPD) clinics in two regional referral hospitals and six health centres in six regions in Tanzania. Methods: The patients were consecutively recruited, and the data collection was conducted in eight health care facilities from 2014 to 2016. Genital swabs were collected for the detection of the aetiological pathogens of STI/RTIs. Results: A total of 1243 participants were recruited in the study; the majority (1073, 86%) were women. The overall median age was 27.8. The prevalence of *Neisseria gonorrhoeae* was 25.7% (319/1243), with proportions of 50.9 and 21.5% for men and women, respectively, of *Chlamydia trachomatis* 12.9% (160/1241) and *Mycoplasma genitalium* 4.7% (53/1134). Unmarried men were more often likely to be infected with gonococcal infections as compared to their women counterparts (57.9 vs. 24.1%) *p* < 0.001. The majority presented with genital discharge syndrome (GDS) 93.6% (1163/1243), genital ulcer disease (GUD) 13.0% (162/1243) and GDS + GUD 9.6% (119/1243). GDS was more common in the health centres, 96.1% (1195/1243), vs. the regional referral hospitals, 92.2% (1146/1243) (*p* = 0.01), but those reported to the regional referral hospitals were more likely to be infected with *N. gonorrhoeae* (OR = 2.5) and *C. trachomatis* (OR = 2.1) than those from the health centres (*p* < 0.001). The prevalence of bacterial vaginosis (BV) and vaginal candidiasis (VC) was 24.1 and 10.4%, respectively. Interestingly, unmarried and BV-positive women were less likely to be infected with VC (*p* = 0.03), though VC was strongly inversely associated with an *N. gonorrhoeae* infection (*p* < 0.001). High proportions of *N. gonorrhoeae* (51.1%) and *C. trachomatis* (23.3%) were found in the Dodoma and Dar es Salaam regions, respectively. *M. genitalium* (7.6%) was found to be the highest in Mwanza. Conclusion: We reported a high prevalence of STI/RTIs. The findings suggest that these infections are common and prevalent in STI/OPD clinics in six regions of Tanzania. We recommend surveillance to be conducted regularly to elucidate the true burden of emerging and classical STI/RTIs by employing modern and advanced laboratory techniques for the detection and monitoring of STI/RTIs in low- and high-risk populations, including the community settings.

## 1. Introduction

Sexually transmitted infections (STIs) are commonly caused by diverse aetiological microorganisms, which include bacterial, viral, fungal and or parasitic infections. Most of these microorganisms cause high morbidity with serious complications and consequences, such as miscarriages, postpartum sepsis, foetal wastage and pelvic inflammatory disease (PID), with which it greatly contributes to high rates of infertility, maternal and perinatal morbidity and mortality [1]. STIs are a major public health problem worldwide [2]. Recent reports indicated that 273 million prevalent cases of STIs occur annually among people aged between 15 and 49 years [1,3]. The incidence and prevalence are common in the world, particularly in the resource-constrained countries [1,3,4].

In resource-constrained countries, the rates of gonorrhoea and chlamydia remain high, and the underestimates thereof are also high because of ineffective diagnostic methods and inaccessibility to health care facilities. In 2008 to 2013, previous studies reported varying rates of 0.3 to over 20% for gonorrhoea in the large cities of Dar es Salaam and Mwanza (Tanzania) and in Lusaka (Zambia) among women attending STI/outpatient department (OPD) and antenatal clinics [5,6].

*Mycoplasma genitalium* is a causative agent of non-gonococcal non-chlamydial urethritis (NGNCU) in men and cervicitis in women, and it is reported to be associated with PID, infertility and preterm birth [7]. *M. genitalium* was first isolated in the early 1980s in men with NGNCU, but owing to the difficulties in detecting the microorganism by culture, much research was conducted since the development of nucleic acid amplification tests. In population- and health care-based studies, *M. genitalium* was detected in substantial proportions of men with urethritis and women with cervicitis. Based on these studies, routine testing was suggested to detect and treat *M. genitalium* in symptomatic attendees in health care settings, and the recommendation was also extended to low-risk general populations [7,8,9,10].

Bacterial vaginosis (BV) and vaginal candidiasis (VC) are essentially non-sexually transmitted reproductive tract infections (RTIs) which are often the main reason for women to present with genital complaints to most health centres. In both cases, there is much inconsistency in the diagnostic algorithms used, and the data also show little consistency. It is clear, however, that the prevalence of BV is extremely high, with the highest prevalence rates being observed in both health care settings [6,11,12] and the community population-based studies [12,13,14]. VC, which is usually characterised with curd-like discharge among women attending STI/OPD clinics, was reported to have a high prevalence. The typical symptoms include pruritus, vaginal soreness and or dyspareunia. It is estimated that 75% of women will have at least one episode of this disease and 40–45% will have two or more episodes [4].

The epidemiology of STI/reproductive tract infections (RTIs) is influenced by many interrelated factors including socio-geographical which produce multiple micro epidemics [2]. The factors influencing the high prevalence of these infections are not only found in lower socioeconomic groups [15] but also in higher socioeconomic groups, particularly among different populations, such as men who have sex with men (MSM), international travellers, commercial sex workers and their clients [3,4]. Young people who initiate sex early in adolescent pregnant women are as well among those who are at increased risks of gonorrhoea, chlamydia and BV [16]. Multiple infections ranging from 2 to 40% of gonorrhoea, chlamydia and BV in different populations were also reported in previous studies [5,17,18].

The effective intervention and control of these infections using the syndromic approach hinges on the understanding and monitoring of current, local and international patterns of aetiological pathogens [1,12]. In resource-constrained countries including Tanzania, syndromic management is being implemented, and though this is a cost-effective strategy, its value is very limited not only in the detection and management of asymptomatic infections but also because symptomatic populations receive inappropriate treatment or overtreatment. Hence, recurrent episodes among symptomatic patients are currently escalating, resulting in the overburden of health care services. Recurrent episodes can be associated with high multiple/mixed infections and sometimes treatment failure to common genital tract infections is high due to drug resistance [12,19].

Many previous studies reported that most patients, especially women, that were diagnosed by the syndromic approach revealed that their total infection load as determined by aetiological diagnosis is quite low. This could mean that these infections are being over-diagnosed by the investigating physicians and even that physiological discharge is misinterpreted as pathological. For example, a previous study found that psychosocial factors instead of aetiological agents were found to have the strongest association with the complaint of genital discharge, especially in women. It was recommended that syndromic management algorithms be refined so that women with complaints that are non-infectious in aetiology are offered psychosocial interventions [13].

The early detection and monitoring of emerging and classic aetiological pathogens of STI/RTIs require advanced laboratory expertise, especially in resource-constrained countries. Most clinical laboratories lack the ability and expertise to conduct microbiological testing for the detection of common infections. In Tanzania, the current and available data on the aetiological pathogens of STI/RTIs are limited. However, there is a need for the current data to guide management and inform the regular review and update of the syndromic management approach as recommended by the WHO [1,20].

Therefore, determining current aetiological pathogens of STI/RTIs using reliable and advanced modern molecular techniques is pivotal in order to generate current and correct information to appraise and review the treatment guidelines for the effective management and control of these infections in the country. In the present study, we determined the prevalence of common STI/RTIs caused by *N. gonorrhoeae, C. trachomatis, M. genitalium*, *Gardnerella vaginalis and Candida albicans*. Further, the study aimed at generating current information on the magnitude and prevalence of the aetiological agents and risk factors associated with STI/RTIs from patients presenting with genital symptoms in two regional referral hospitals (Dodoma and Mwanza) and six health care facilities (Dar es Salaam, Mwanza, Kilimanjaro, Tanga and Mbeya) among six geographical regions in Tanzania. Furthermore, the performance characteristics of syndromic management algorithms compared to laboratory techniques were also evaluated.

## 2. Materials and Methods

### 2.1. Study Design and Population

This was a cross-sectional, health facility-based study whereby patients who attended STI/OPD clinics presenting with genital symptoms were recruited between 2014 and 2016. Patients who presented with genital discharge syndrome (GDS) and/or genital ulcer syndrome (GUS) symptoms were recruited; socio-demographic and clinical data were consecutively collected at one point in time at each study site. Eight health care facilities in the six regions that were randomly selected from a total of sixteen clinics were Infectious Disease Clinic (IDC), Dar es Salaam; Ngamiani Health Centre, Tanga; KRHP, Kilimanjaro; Igawilo and Ruanda health centres, Mbeya; regional referral hospital, Dodoma and Sekou Toure regional referral hospital; and Igoma health centre, Mwanza. After obtaining written informed consent, participants were recruited and enrolled in each study site. However, written assent was sought and obtained from a parent or a guardian for a participant aged 15 years old (approvals by the National Institute for Medical Research Ethics Committee Ref. No. NIMR/HQ/R.8c/Vol.1/191 and MUHAS Research and Publications Committee Ref. No. 2016-06-07/AEC/Vol. XI/28). Adult participants who did not consent to participate in the study and a parent or a guardian who did not provide assent for 15 year old were excluded from the study.

GDS was defined as self-reported or having a discharge (examined). The presence of a vaginal discharge (VD) with either mucopurulent, yellowish-green, curd-like, lower abdominal pain/low backache, itching and/or redness was noted and recorded during clinical examination and the presence of urethral discharge (UD) was noted after “milking” the urethra if a discharge was not initially seen. GUS was defined as a reported and/or examined presence of an ulcer, sore, vesicle, genital rash and or warts on examination.

### 2.2. Data Collection and Laboratory Methods

An interview was conducted using a structured questionnaire. Genital examination was performed, and specimen was collected by a qualified and trained clinician who was attending the patient. A dacron swab was collected from endo-cervical and urethral exudates for detection of common pathogens of STIs. Another swab was collected from the vaginal wall. *N. gonorrhoeae* was identified by colonial morphology, Gram stain and oxidase test. Further, *N. gonorrhoeae* isolates were confirmed by Real-Time PCR assay.

A smear was made and Gram stained for Nugent score for the detection of BV and the same smear was also used for microscopic examination of Gram-positive yeast, hyphae or pseudohyphae for detection of VC. Urethritis and/or cervicitis was defined as the presence of five or more polymorphonuclear leucocytes per high power field (PMNs ≥5/HPF) in the urethral/vaginal smear. Specimens to be used to test for the presence of *N. gonorrhoeae*, *C. trachomatis* and *M. genitalium* were transported in a cool box and stored at −20 to −40 °C before testing by Artus CT/NG QS-RGQ real-time Polymerase Chain Reaction (PCR) assay (Qiagen GmbH, Hilden, Germany). Smears for detection of BV and VC by Nugent Score were heat fixed and transported in a slide box and stained by Gram-stain technique. The most common Amsel’s criteria and the gold standard laboratory-based Nugent Gram-staining evaluation were used (21, 22). Slides of vaginal smears were Gram stained and the bacterial morphotypes were quantified and scored as follows: Large Gram-positive rods (Lactobacillus scored as 0 to 4), small Gram-variable rods (*Gardnerella vaginalis* scored as 0 to 4) and curved Gram-variable rods (Mobiluncus species scored as 0 to 2). Bacterial vaginosis was put on a 10-point scale where 0–3 was regarded as normal (predominantly Lactobacillus), 4–6 as intermediate (mixed flora) and 7–10 positive for BV (no Lactobacillus).

### 2.3. DNA Extraction and Testing

Buffers in the reagent cartridge were checked if they did not contain a precipitate (Qiagen GmbH, Hilden, Germany). Troughs containing buffers from the reagent cartridge were removed and incubated for 30 min at 37 °C with occasional shaking to dissolve the precipitate (Qiagen GmbH, Hilden, Germany). When the reagent cartridge was already pierced, the troughs were sealed with Reuse Seal Strips and complete reagent cartridge was incubated for 30 min at 37 °C with occasional shaking in a water bath. Bubbles from the surface were aspirated and the buffer was left to cool to 15–25 °C. Vigorous shaking of the reagent cartridge was avoided to generate foam which could lead to liquid-level detection problems. Swabs were suspended in phosphate-buffered saline, urogenital materials and cells were pelleted by centrifugation. DNA was extracted in an alkaline lysis buffer and bound onto the QIAamp silica-gel membrane during a brief centrifugation (Qiagen GmbH, Hilden, Germany). Purified DNA was eluted from the QIAamp spin column in a concentrated form in a Tris buffer.

### 2.4. Real-Time PCR Assays

Real-time PCR for qualitative detection of *C. trachomatis* plasmid and *N. gonorrhoeae* genomic DNA was performed using Artus CT/NG QS-RGQ real-time PCR assay (Qiagen GmbH, Hilden, Germany). The components of the *artus* CT/NG QS-RGQ assay were stored at –15 to –30 °C and were stable until the expiration date. Repeated thawing and freezing more than two times was avoided as this might have reduced performance of the assay. If the reagents were to be used only intermittently, they were frozen in aliquots. Storage at 2–8 °C did not exceed 5 h. Before each use, all reagents were thawed completely, mixed by quick vortexing and centrifuged for at least 3 s at 6800× *g*. All reagents that were loaded on the assay setup module were used in that run only. The CT/NG RG Master contained reagents and enzymes that were for the specific amplification of 86 and 66 bp regions of the *C. trachomatis* genome and a 74 bp target of the *N. gonorrhoeae* genome. Real-time PCR was also used for detection of *M. genitalium.* Target amplification and detection were performed in a Rotor-Gene Q instrument (Qiagen, Hilden, Germany). The instrument was checked, maintained and calibrated according to manufacturer’s instructions. All processes from DNA extraction, amplification and detection were conducted according to manufacturer’s instructions (Qiagen, Hilden, Germany). Each assay included internal controls (ICs) and external positive and negative controls (Control CT+/NG− and Control NG+/CT−). Testing was performed at NIMR Mwanza laboratory and results were verified and reported as per manufacturer’s instructions.

### 2.5. Data Analysis

Socio-demographic and laboratory data were entered into CSPro software (https://www.census.gov, accessed on 19 October 2022) for data analysis. Prevalence of STI/RTIs and associated factors were determined, and data analysis was performed using Epi Info 7.0 (http://wwwn.cdc.gov/epiinfo/7/index/htm, accessed on 19 October 2022) and STATA 13.0 (Stata Corp, Timberlake, NC, USA). Chi square and t-tests where applicable were performed to assess the associations of different aetiological pathogens of STI/RTIs and the associated factors. In logistic regression analysis, intermediate category of Nugent Score was excluded in the analysis for BV. Sensitivity, specificity, PPV and NPV analyses of syndromic management were also performed. Findings were presented as odds ratio (OR) at 95% CI. A *p* value of <0.05 was regarded as statistically significant. *p* values exceeded or ≤0.000 were truncated to <0.001.

## 3. Results

### 3.1. Socio-Demographic Characteristics

A total of 1243 participants were included in the study. Women (1073, 86.3%) consisted of the majority of the study participants. The overall median age was 27.8 (range 15–78 years) and the mean ratio of M:F was >6, indicating that women with genital symptoms were more likely to present to health care facilities than men. Over 80% of all the participants were in the age group between 15 and 45 years.

### 3.2. Prevalence of and Factors Associated with STI/RTIs

The prevalence of *N. gonorrhoeae* was 25.7% (320/1243), with proportions of 50.9 and 21.5% observed among men and women, respectively. Moderate and low rates of 12.9% (160/1241) and 4.7% (53/1134) were found for *C. trachomatis* and *M. genitalium*, respectively. The highest proportions of 51.1% (163/320) and 46.9% (75/160) for *N. gonorrhoeae* and *C. trachomatis* were observed among patients who presented to the referral health facilities in the Dodoma and Dar es Salaam regions, respectively. *M. genitalium* was the highest (7.6%; 4/53) among patients reported at the Sekou Toure regional referral hospital and the Igoma health centre in the Mwanza region.

The prevalence of BV was 24.1% (212/883), with the highest proportion of 31.6% (67/212) found in Moshi, Kilimanjaro, among patients attending a reproductive health program. Using the Nugent Score, BV-positive women were classified into three main categories, those who had BV attributed only to the *Gardnerella vaginalis/Bacteroides*/*Prevotella* species (79.2%), *G. vaginalis/Bacteroides*/*Prevotella* plus *Mobiluncus* (17.8%) and *Mobiluncus* species (3%). The prevalence of VC was 10.4% (107/1032). There was a statistically significant univariate association among women who had BV compared to those who had VC (*p* = 0.02). Women who had BV were more likely to be infected with an *N. gonorrhoeae* infection (*p* < 0.001) compared to those who were uninfected. Women infected with VC had consistently exhibited a significant association with an *N. gonorrhoeae* infection (*p* < 0.001).

The overall prevalence (total infection load) of the infections attributed to STIs and RTIs was 44.7% (555) and 33.3% (345), respectively. The proportions of all the STIs were 35.1, 8.6 and ~1% for single, double and >3 multiple infections, respectively. Unmarried men were more likely to be co-infected by either *N. gonorrhoeae*, *C. trachomatis* and/or *M. genitalium* than their women counterparts (57.9 vs. 24.1%; *p* < 0.001).

For symptoms, the majority of the participants, (93.6%; 1163/1243) presented with GDS, GUD (13.0%; 162/1243) and GDS + GUD (9.6%; 119/1243) reported, to present either singly or in combination with other clinical symptoms during the clinical history and genital examination procedures. Aetiologically, of all those who presented with GDS and GUD, only 4.7 to 25.7% and 1.8 to 9.6% were found infected with either *C. trachomatis, N. gonorrhoeae* and/or *M. genitalium,* respectively. The proportions observed among men with urethritis and women with cervicitis accounted for 45.6 and 39.3%, respectively.

In the logistic regression analysis models (Table 1), the socio-demographic and clinical data were compared specifically with the laboratory results to determine the association of these infections. Men were more likely to be infected with *N. gonorrhoeae* than women (adjusted OR = 3.7). In the model, overall (adjusted OR = 3.7) the *N. gonorrhoeae* infection was significantly and consistently found associated with many risk factors as adjusted to age, marital status, type of health facility, study site (region) and type of syndromes. This indicates that *N. gonorrhoeae* strains were diversely and heterogeneously associated with many infections among different risk factors. The patients who presented to the referral hospitals had higher risks of being infected with *N. gonorrhoeae* (AOR = 2.5) and *C. trachomatis* (AOR = 2.1) than those who presented to health centres (*p* < 0.001). However, those who presented with GDS were more often likely to be infected with *C. trachomatis* (*p* = 0.01) and those with GUD were less likely to be infected with *N. gonorrhoeae* (*p* = 0.006) than any other infections, respectively.

The genital syndromes were compared with the laboratory results to determine the performance characteristics for the detection of STI/RTIs using the syndromic management strategy. A low specificity (6.9%) and poor predictive values (PPV) 25.8% were obtained when GDS was compared with the laboratory results for the detection of *N. gonorrhoeae* infection (Table 2). GDS was poorly predictive and unspecific of a laboratory diagnosis for *N. gonorrhoeae* especially in women (*p* = 0.49). In the microscopy, we reported a low PPV of 28.0%, *N. gonorrhoeae;* 16.2%, *C. trachomatis;* and 6.3%, *M. genitalium* in relation to the detection of urethritis and/or cervicitis among men and women in this population.

## 4. Discussion

This was a cross-sectional health facility-based study which was conducted in referral hospitals and health centres among patients who presented with genital symptoms to STI/OPD clinics in six regions of Tanzania. The overall prevalence (total infection load) of the STI/RTIs was very high and these infections were common and prevalent across the study sites. These findings suggested an indication that this high prevalence and the common infections found in this population could also be similar or quite high in some community settings in Tanzania.

Confirming our report, the high prevalence rates of STI/RTIs were also documented by many previous studies conducted among different populations in sub-Saharan Africa, including Tanzania and in India [5,6,11,12,18,21,22,23]. However, the reasons for the increase in these infections are unclear. The present study has elucidated some possible explanations for the increase in these infections. First, the lack of effective screening, surveillance and intervention programs for early detection, regular monitoring and control of STI/RTIs among different populations could have resulted in high proportions of infections for individuals who may have been exposed to and/or had symptoms/signs at either an earlier stage or later. Due to the natural history of untreated or inadequately treated infection, especially for gonorrhoea and/or chlamydia, a symptomatic illness can then be followed by a protracted spell of asymptomatic infection, especially in women, and thus these individuals remain as an infective pool in the general population. Hence, they become an important source of transmission of new infections to their counterparts (low to high risk communities) until they develop signs and symptoms which require them to seek medical attention in referral and or primary health care settings.

Unlike in developing countries, there is a great emphasis on the importance of sustained public health programmes to identify and treat infections, reduce morbidity and mortality and prevent onward transmission. Many reports from both population- and clinic-based estimates indicate that low rates of <3% for classic STIs such as gonorrhoea and chlamydia are frequently reported [1,2,9,13], except for core groups, such as MSM and other people who work in prostitution facilities such as brothels, bars and nightclubs [16]. However, in developing countries, especially in sub-Saharan Africa, the prevalence of these infections is consistently reported as high among low- and high-risk populations [5,6,7,11,14,21,22,24] because of the limited resources to conduct regular, effective screening and surveillance programs.

Previous studies still reported high rates of genital infections among different populations [5,6,11]. In a previous study that was conducted to determine STI/RTIs among HIV-infected pregnant women in Moshi, Tanzania, the prevalence of BV was 37.2% [5]. A previous study conducted among women attending STI clinics in Mwanza, Tanzania, showed a prevalence of 8.4, 14, 25.9 and 25.6% for *N. gonorrhoeae*, *C. trachomatis* infections, BV and VC, respectively [6]. Previously published studies of antenatal clinic attendees in Africa also documented that the prevalence of gonorrhoea and chlamydia ranged from 2 to 7% and 3 to 29%, respectively, while BV accounted for up to 40% [11]. Data from Tanzania and elsewhere in Africa indicated that a large proportion of patients with STIs either failed to seek treatment or received inadequate treatment, often with inappropriate antimicrobial drugs mostly obtained through commercial outlets [21].

Second, the high rates of infections in the referral hospitals and primary health care settings provided another important reason that symptomatic patients may have higher rates of these infections than the general population. Recruiting symptomatic subjects may have influenced the prevalence of these infections, especially for STIs. This is important because individuals who are recruited in referral hospitals and primary health care facilities living in and around large cities may have been exposed to higher transmission dynamics and thus increase onward transmission among groups. However, this could be one of the limitations that these results may not reflect similar rates in a general population. Nonetheless, our findings remain striking and raise concern that a high prevalence of these infections is common and prevalent among patients attending STI/OPD clinics in Tanzania.

There is clear evidence that high rates of infections are always demonstrated in most studies where there are no ongoing or regular effective intervention programs, in both population- and clinic-based studies, particularly in resource-limited countries [6,14]. In comparison, other recent studies reported high rates of infections among African populations, especially in large cities in sub-Saharan Africa [5,16,19,24,25,26]. A prevalence of 10–25% was reported among different populations in large cities of Mwanza (Tanzania), KwaZulu Natal (South Africa), Harare (Zimbabwe) and Kampala (Uganda) [12,24,27,28].

The increase is also attributed to a complex set of socio-economic dynamics (barriers), including biomedical intervention that requires a broad scope of sensitive and advanced diagnostic tests to reliably and accurately detect specific aetiological pathogens among highly dynamic populations. One study recommended that expanding the regular testing of STIs is another important mechanism for risk reduction strategies for STIs [18] and exploiting the latent demand for STI testing should be integrated into other health care services, such as HIV care services in Tanzania.

However, on the contrary, few studies have also reported low rates or a decline in the prevalence and incidence following screening and intervention programs conducted in reproductive health clinics in Tanzania [11,12,23,25]. Better designed and effective interventions provide better environments for the uptake of health care services by the clients. The present study was conducted in symptomatic patients only accessing routine services at STI/OPD in referral and health centres without intervention programs. The failure to implement effective intervention and control programs as one of the reduction strategies for high infections among many populations may have contributed to the increase in these infections [11].

Furthermore, the present study observed low specificity and poor predictive values obtained when genital syndromes were compared with laboratory results for the detection of these infections. This implies that more false positive cases were given treatment of which they do not need, hence overtreatment, and this impacts a little in reducing the burden of these infections not only in this population but also in other populations. While a high proportion (>90%) of patients were diagnosed by the syndromic approach (GDS), their total infection load as determined by an aetiological diagnosis was low, (26.1%) and (18.3%) for STIs and both mixed STI/RTIs, respectively. This could mean that these infections are being over-diagnosed by the investigating physician and even that physiological discharge was misinterpreted as pathological. Although, the sensitivity of the syndromic approach for GDS in this study was high, but a low specificity was observed as reported previously [12,28].

Multiple infections were also demonstrated in this study. Multiple infections are a reflection of high and common infections across the communities, including low-risk communities. This can be compounded by the inappropriate use of antimicrobial drugs taken without prescriptions by individuals who become symptomatic. Evidence shows that 66.7–100% of women with *N. gonorrhoeae* and 31.2-100% with *C. trachomatis* are asymptomatic [29]. Hence, a large pool of individuals, especially women, live with asymptomatic infections, and these individuals serve as a source of transmissions of new infections to their counterparts [18]. If this is the case, then improved clinical services that ensure that patients, especially men, receive effective treatment soon after the onset of symptoms may have a major public health impact.

In a multivariate analysis model, we have found that *N. gonorrhoeae* was significantly associated with many risk factors which were found to be associated with gonococcal infections. However, the reason was unclear as perhaps the *N. gonorrhoeae* strain possesses genetic adaptive mechanisms that enabled it to be highly diverse and consistently associated with many infections in humans.

This study has reported a low rate of 4.7% for the emerging *M. genitalium* and this was also found to be the highest in the Mwanza region. *M. genitalium* is an emerging agent of non-gonococcal non-chlamydial urethritis (NGNCU) in men and cervicitis in women, and it is reported to be associated with PID, infertility and preterm birth [7]. It is recommended to detect and treat *M. genitalium* in symptomatic attendees in health care settings and the recommendation was also extended to low-risk general populations [7,8,9].

Finally, other infections that involve RTIs were reported to be moderate to high. In most cases, BV is the most prevalent cause of vaginal discharge in developing countries. Up to 50% of women were found to have BV in sub-Saharan Africa [5,6,11,14]. However, in the present study, the prevalence of BV was found to be far decreased. The reason for the decrease is unclear; perhaps metronidazole was introduced into syndromic management [20]. In addition, VC was >10%; this infection is usually treated with less attention because it is not characterised with acute and severe onset. VC is usually between 20 and 40%, except in studies in which definitions are based on a clinical diagnosis [4]. It is usually caused by *C. albicans* but can occasionally be caused by other *Candida* spp. Approximately 10–20% of women will have complicated VC requiring special diagnostic and therapeutic considerations [4]. The study has a limitation. In the logistic regression analysis, an intermediate category of the Nugent Score was excluded in the analysis for BV and this might have underestimated the prevalence of BV.

## 5. Conclusions

We concluded that, in this study, we have reported a high prevalence of STI/RTIs, and these infections were common and prevalent across the study sites. These findings suggest an indication that the high prevalence and common infections found in this population can also be similar or quite high in other community settings in Tanzania. *N. gonorrhoeae* was heterogeneously diverse and highly associated with multiple risk factors than other classic STI/RTIs as determined in this study. This warrants further investigation on the genetic adaptive mechanisms that promote its survival and persistency in humans.

It is important to target both low- and high-risk groups because they continue to be the source for seeding new infections in general populations [23]. We recommend that more and large studies should be conducted in different populations with a broad spectrum of tests for greater coverage to different aetiological agents of STI/RTIs in different populations, especially in low-risk community settings. There is an urgent need to allocate more resources for the control and intervention against the increase in STI/RTIs among different populations, as these infections continue to impact on reproductive health, pregnancy and maternal and perinatal outcomes. Targeted interventions including screening and surveillance programs greatly contribute to the control and monitoring of STIs. Hence, our findings have provided important information for planning and evidence-based decision making in Tanzania.

## Figures and Tables

**Table 1:** Prevalence and Odds Ratios of Risk Factors Associated with STI/RTIs among Study Participants.

Variable	^+^*N. gonorrhoeae* PCR N = 1243	^+^*C. trachomatis* PCR N = 1241	^+^*M.* *Genitalium* PCR N = 1134	* Bacterial Vaginosis Nugent Score N = 883	* Vaginal Candidiasis N = 1032	** *N.* *gonorrhoeae* Culture N = 1243
	n (%) AOR (95% CI)	n (%) AOR (95% CI)	n (%) AOR (95% CI)	n (%) AOR (95% CI)	n (%) AOR (95% CI)	n (%) AOR (95% CI)
Overall prevalence	319 (25.7) 1.9 (1.2–3.3)	160 (12.9) 1.4 (0.7–2.9)	53 (4.7) 1.2 (0.5–4.9)	212 (24.1) 0.8 (0.5–1.7)	107 (10.4) 0.7 (0.3–1.5)	98 (7.9) 2.5 (1.7–3.9)
Sex Men	Reference					
Women	162 (50.9%) 3.7 (2.7–5.2)	17 (10.6) 0.7 (0.4–1.2)	3 (5.5) 1.0 (0.5–1.5)	53 (24.8) 0.6 (0.3–1.4)	14 (12.8) 0.3 (0.1–1.5)	29 (29.9) 4.5 (3.7–8.2)
Age, years 15–35	Reference					
36–55	78 (24.6%) 0.9 (0.2–3.3)	20 (12.5) 1.6 (0.6–4.3)	2 (3.6) 1.2 (0.3–1.8)	57 (26.8) 0.6 (0.3–1.5)	26 (24.0) 0.5 (0.3–1.8)	6 (6.4) 2.5 (1.1–7.7)
56–78	91 (28.4%) 1.1 (0.3–4.0)	25 (15.3) 1.8 (0.6–5.1)	2 (2.9) 0.5 (0.9–1.2)	60 (28.5) 0.7 (0.2–2.9)	-	-
Marital status Not married	98 (30.7%) 1.8 (0.6–5.3)	21 (12.9) 0.9 (0.6–1.2)	3 (5.1) 1.2 (0.5–3.1)	48 (22.4) 0.8 (0.6–1.0)	10 (8.9) 0.6 (0.4–1.0)	9 (9.1) 1.9 (1.2–2.9)
Residence Rural	Reference					
Urban	97 (30.4%) 1.5 (1.2–2.0)	26 (16.3) 2.1 (1.4–3.1)	3 (6.1) 1.3 (0.5–3.2)	50 (23.7) 0.8 (0.5–1.1)	12 (11.2) 0.7 (0.6–1.4)	7 (6.8) 1.0 (0.6–1.6)
STI/RTI knowledge No	Reference					
Yes	100 (31.4) 2.5 (1.8–3.3)	20 (12.2) 1.0 (0.6–1.6)	3 (5.3) 1.4 (0.4–4.4)	51 (24.0) 0.9 (0.6–1.3)	10 (9.4) 0.7 (0.4–1.2)	8 (8.0) 1.2 (0.7–2.1)
Had STIs 12 months ago No	Reference					
Yes	76 (23.9) 1.0 (0.7–1.4)	19 (12.0) 1.4 (0.9–2.1)	3 (5.2) 1.3 (0.5–3.4)	52 (24.3) 0.9 (0.6–1.1)	13 (12.1) 1.1 (0.7–1.7)	7 (6.7) 0.8 (0.5–1.3)
Treated STI/RTI before No	Reference					
Yes	85 (26.7) 1.5 (1.1–2.1)	30 (18.9) 1.2 (0.7–2.1)	4 (7.9) 2.0 (0.8–5.4)	55 (26.0) 1.0 (0.6–1.6)	11 (10.0) 0.6 (0.3–1.1)	6 (6.5) 1.3 (0.5–1.3)
Presented GDS only No	Reference					
Yes	82 (25.8) 1.2 (0.7–2.1)	22 (13.9) 2.9 (1.1–8.7)	3 (5.2) 1.3 (0.4–4.4)	53 (25.1) 1.2 (0.6–1.6)	12 (11.1) 0.7 (0.3–1.5)	6 (6.5) 1.3 (0.5–3.0)
Presented GDD only No	Reference					
Yes	57 (17.9) 0.5 (0.3–0.9)	16 (9.8) 0.6 (0.2–1.1)	3 (4.8) 0.9 (0.4–2.1)	47 (22.3) 0.8 (0.5–1.3)	7 (6.7) 0.5 (0.2–1.0)	7 (6.6) 0.8 (0.3–1.6)
GDS + GUD No	Reference					
Yes	58 (18.2) 0.9 (0.3–0.9)	18 (11.1) 0.7 (0.4–1.4)	1 (1.8) 0.3 (0.1–1.3)	60 (28.3) 1.2 (0.7–1.8)	8 (7.1) 0.5 (0.2–1.1)	7 (7.1) 1.1 (0.5–2.2)
PMN <5/HPF	Reference					
PMN >5/HPF	89 (28.0) 1.2 (0.9–1.6)	26 (16.2) 1.1 (1.1–2.1)	3 (6.3) 1.5 (0.8–2.5)	49 (23.2) 0.8 (0.6–1.0)	16 (15.2) 1.8 (1.2–2.7)	10 (10.4) 2.6 (1.7–4.2)
Region Mbeya (HCs)	42 (13.3) 1.8 (0.6–5.3)	14 (8.9) 0.3 (0.1–2.0)	2 (3.1) 1.0 (0.6–1.8)	35 (16.3) 1.0 (0.7–2.1)	8 (7.3) 1.0 (0.6–2.5)	7 (7.0) 1.0 (0.4–1.7)
Dar es Salaam (IDC)	129 (40.5) 4.4 (2.7–5.3)	20 (12.3) 1.8 (0.9–3.5)	3 (4.6) 1.2 (0.4–3.9)	25 (11.8) 1.0 (0.5–1.0)	10 (9.0) 0.8 (0.7–1.0)	13 (12.9) 4.4 (2.7–7.5)
Dodoma RRH	163 (51.1) 4.7 (2.9–6.3)	37 (23.2) 3.1 (1.6–5.8)	3 (5.6) 1.8 (0.6–5.4)	50 (23.4) 1.6 (0.3–3.4)	16 (15.0) 1.6 (0.3–3.1)	1 (1.4) 1.2 (0.5–4.1)
Mwanza RRH	41 (12.9) 1.4 (0.7–2.0)	21 (12.9) 1.5 (0.8–2.8)	4 (7.5) 2.6 (1.0–7.3)	51 (24.1) 0.3 (0.1–0.7)	16 (15.0) 0.3 (0.1–0.8)	7 (6.7) 1.4 (0.3–2.7)
Tanga (HC)	52 (16.4) 1.2 (0.6–2.1)	13 (7.9) 1.0 (0.4–2.0)	2 (4.6) 1.5 (0.5–4.4)	53 (25.1) 0.8 (0.3–2.0)	7 (6.6) 0.3 (0.1–2.1)	7 (6.8) 1.5 (0.5–3.2)
Kilimanjaro HC)	60 (18.8) 1.5 (0.7–3.2)	7 (4.3) 0.4 (0.2–1.5)	1 (1.4) 0.4 (0.1–3.9)	67 (31.6) 0.9 (0.5–2.4)	7 (6.7) 0.9 (0.5–2.2)	4 (4.3) 1.5 (0.7–3.2)

+ = Number of participants varies according to the test performed: NG = *N. gonorrhoeae* (N = 1243), CT = *C. trachomatis* (N = 1241), MG = *M. genitalium* (N = 1134). * = RTIs = BV = Bacterial vaginosis (N = 883), VC = Vaginal candidiasis (Candida species) (N = 1032), (RTIs = Detection of BV and VC was only performed in women). ** = *N. gonorrhoeae* (Culture) = Gold standard.

**Table 2:** Performance Characteristics of Syndromes as Compared with Laboratory Results for Detection STI/RTIs.

Test	(A) *N. gonorrhoeae*		(PCR)		(B) *N. gonorrhoeae*		(Culture) **	
Syndrome	Sensitivity (%)	Specificity (%)	PPV (%)	NPV (%)	Sensitivity (%)	Specificity (%)	PPV (%)	NPV (%)
1. GDS Only	(94.3)	(6.9)	(25.8)	(78.1)	(90.3)	(6.2)	(6.5)	(90)
2. GUD Only	(9.1)	(85.6)	(17.9)	(73.3)	(10.8)	(86.8)	(5.6)	(93.1)
3. GDS + GUD	(7.3)	(88.9)	(18.2)	(73.6)	(10.8)	(89.9)	(7.1)	(93.4)
4. Microscopy	(44.1)	(61.1)	(28.0)	(76.1)	(61.2)	(62.6)	(10.4)	(95.8)
	**(C) *C. trachomatis***		**(PCR)**		**(D) *M. genitalium***		**(PCR)**	
1. GDS Only	(97.6)	(7.3)	(13.9)	(95.1)	(94.8)	(6.9)	(5.2)	(96.1)
2. GUD Only	(9.7)	(86.4)	(9.8)	(86.1)	(12.1)	(87.1)	(4.8)	(94.8)
3. GDS + GUD	(8.5)	(89.6)	(11.1)	(86.4)	(3.4)	(89.9)	(1.8)	(94.5)
4. Microscopy	(49.1)	(61.1)	(16.2)	(88.7)	(48.3)	(61.2)	(6.3)	(95.6)
	(E) Bacterial Vaginosis *		**(Nugent Score)**		(F) Vaginal Candidiasis *		**(Gram stain)**	
1. GDS Only	(94.9)	(6.3)	(25.1)	(79)	(92.3)	(5.8)	(11.1)	(85.4)
2. GUD Only	(11.7)	(86.6)	(22.4)	(74.8)	(7.7)	(86.3)	(6.7)	(87.9)
3. GDS + GUD	(12.4)	(89.5)	(28.3)	(75.6)	(6.8)	(88.5)	(7.1)	(88.1)
4. Microscopy	(37.4)	(59.2)	(23.2)	(74.1)	(53.8)	(61.8)	(15.2)	(91.3)

* RTIs = detection was performed only in women, ** = N. gonorrhoeae (culture) = gold standard.

## Data Availability

We collected demographic and clinical information from all participants; these raw data were generated and analysed in the Data Management Unit (DMU) at the NIMR Mwanza Centre. The datasets of this study are available and deposited in the local public domain of the NIMR data server. The generated datasets will be made available to the editor on request from the corresponding author or the director of the NIMR Mwanza Centre.
